# Burnout Risk Among Providers of an Integrated Care Program Supporting Transitions Between the Hospital and Home: A Descriptive Mixed Methods Evaluation

**DOI:** 10.3390/ijerph23050612

**Published:** 2026-05-05

**Authors:** Juma Orach, Aysha Afzaal, Aman Bathla, Zhenxiao Yang, Lauren Lapointe-Shaw, Ceara Cunningham, Valeria E. Rac, Shoshana Hahn-Goldberg, Melissa Chang, Christopher Chan, Carolyn Gosse, Emily Hay, Thomas E. MacMillan, Michelle Grinman, Karen Okrainec

**Affiliations:** 1 Toronto General Hospital Research Institute, University Health Network, Toronto, ON M5G 2C4, Canada; juma.orach@uhn.ca (J.O.);; 2 Department of Medicine, University of Toronto, Toronto, ON M5S 3H2, Canada; 3 Primary Care Alberta, Edmonton, AB T5J 2B8, Canada; 4 NORC Innovation Centre, University Health Network, Toronto, ON M6G 1A5, Canada; 5 Leslie Dan Faculty of Pharmacy, University of Toronto, Toronto, ON M5S 3M2, Canada; 6 Alberta Health Services, Calgary, AB T5J 3E4, Canada

**Keywords:** integrated care, occupational burnout, healthcare providers, care coordinators, homecare providers, integrated care leads, collaborative care

## Abstract

**Highlights:**

**Public health relevance—How does this work relate to a public health issue?**

- Integrated care programs supporting hospital-to-home care transitions improve patient health outcomes and reduce potentially avoidable hospital readmissions through tailored, timely and complex care plans that coordinate home and community services.
- Occupational burnout, which is linked to poor health, reduced patient care quality and high healthcare provider turnover, is relatively understudied for practitioners working on integrated care programs.

**Public health significance—Why is this work of significance to public health?**

- Evaluating burnout and understanding the demographic and workplace factors associated with burnout is paramount for implementing and sustaining integrated care programs.
- We evaluated burnout among healthcare providers of an integrated care program supporting hospital-to-home care transitions, elucidating the promoting and mitigating factors for burnout.

**Public health implications—What are the key implications or messages for practitioners, policy makers and/or researchers in public health?**

- Burnout risk in our sample of integrated care providers was low to moderate—emotional exhaustion was low, depersonalization was moderate, and personal achievement was high, with respondents citing effective workload management, under-appreciation and positive impact on patients as relevant contextual factors respectively.
- Integrated care programs ought to support staff and manage workloads to sustain the positive impact on patients, which in turn confers a sense of accomplishment to healthcare providers.

**Abstract:**

Integrated care programs (ICPs) are associated with positive patient experiences, but provider experiences remain understudied. We examined burnout in healthcare providers working in an ICP that facilitates hospital-to-home care transitions for patients. We conducted a mixed-methods evaluation comprising a cross-sectional survey of burnout and provider experience using the Maslach Burnout Inventory, open-ended questions, and semi-structured interviews. Twenty-eight participants completed the surveys (31% response rate). Respondents were 75% female, and, on average, were 42 ± 10 years old, had spent 19 ± 11 months as providers in the ICP and had cared for a median of 170 (IQR = 245) patients. Twenty staff, who were 38 ± 8 years old on average and 95% women, were interviewed. Emotional exhaustion was low (average total score = 14 ± 7 out of 42), depersonalization was moderate (9 ± 6 out of 42), and personal achievement was high (40 ± 5 out of 48), corresponding to low-to-moderate burnout. Respondents cited teamwork as the leading protective factor against emotional exhaustion and positive impact on patients as the leading factor underlying high personal achievement. Perceived under-appreciation was the leading factor for depersonalization, likely moderated by team support and rapport. Burnout was low to moderate in our sample of ICP healthcare providers, who cited several important contextual factors requiring further study.

## 1. Introduction

The growing prevalence of aging and multimorbidity globally [1,2] has created a need for integrated models of care to address complex care needs and frailty in and outside the hospital [3,4]. Patients living with multiple chronic diseases are among the highest users of the health system with the poorest outcomes [5,6], including a high rate of readmission due to fragmented health services that are ineffective at transitioning patients from the hospital to home [4,7]. Integrated care programs (ICPs) address this care gap by linking various healthcare providers and services in the hospital and/or community to meet the unique care needs of patients, such as managing chronic health conditions and exacerbations [3,8]. These programs have been linked to positive patient experiences as well as reductions in hospital usage and costs [8,9,10]. ICPs rely on healthcare providers for personalized care planning, coordination and service delivery [8,11,12]. Therefore, positive provider experience, which is one of the quadruple aims for health system improvement [13,14], is vital for sustaining ICPs and their impact.

Integrated care for hospital-to-home care transition shifts the care planning tasks from multiple yet sometimes siloed acute and home care providers to one care coordinator, such as an integrated care lead (ICL), to link the inpatient care team to the home or community care team and ensure a seamless care transition [8,11,15]. ICLs are regulated health professionals focused on the patient care experience, including ensuring well-supported discharge planning—they work as one team with homecare service providers, such as nurses, dieticians, personal support workers, social workers, physiotherapists and other community care providers to meet the specific needs of each patient [8,11]. ICPs have been expanding internationally in the last 10 years due to growing demand for home and community care and pressures on acute care to discharge patients home once medically stable [8,16]. With increasing prevalence and recognition of medical and social patient complexity, there is even more pressure for integrated care staff to meet demands for larger and more complex caseloads, take on more administrative tasks, and collaborate in larger teams in resource-constrained settings [8,17]. Such extended periods of intense involvement with patients’ complex social, physical and emotional needs can increase the risk of burnout [17,18].

According to the multidimensional theory of burnout posited by Maslach and Leiter, burnout is a psychological syndrome characterized by (1) emotional exhaustion—feelings of overextension, (2) depersonalization—callousness and cynicism towards care recipients and (3) personal achievement—feelings of efficacy at work [19]. The literature on burnout among care transition coordinators is sparse and findings on burnout in integrated care settings are mixed [20,21,22,23]. This may be explained by organizational, demographic, and role-specific factors [24], but these have not been routinely surveyed and linked to burnout in integrated care for hospital-to-home care transitions [25].

Evaluating burnout and understanding the demographic and workplace factors associated with it is paramount for implementing and sustaining ICPs. Therefore, we used a mixed-methods analysis grounded in this multidimensional theory to explore burnout in a cross-sectional sample of providers of an ICP supporting hospital-to-home care transitions, and identified its promoting and mitigating factors.

## 2. Materials and Methods

### 2.1. Setting

University Health Network (UHN) is a 1322-bed acute care institution in Toronto that delivers 459,890 inpatient days, 1,186,077 outpatient clinic visits and 116,003 Emergency Department visits annually across 7 sites [26]. Inspired by the Ministry of Health Ontario’s Integrated Funding Models [27], UHN created an ICP in 2019 in alignment with the Ontario Health Quality Standards on Transitions of Care [28]. The ICP at UHN was created in collaboration with patients to support care transitions through discharge planning and coordination of health and social services for the 90-day period post-discharge [11]. The ICP achieves this through 4 major tenets across multiple clinical (surgical and medical) pathways: (1) proactive identification of patients with complex care needs or vulnerability, (2) continuity of care through attachment to an ICL who coordinates the patient care plan, (3) timely check-in calls and access to a 24/7 phone line and (4) an integrated care fund to address care transition needs like equipment rentals [29]. At the time of this evaluation, the ICP employed 90 patient care staff, including 18 ICLs and 72 homecare providers across Toronto Western Hospital, Toronto General Hospital and community practice.

### 2.2. Project Design

We conducted a cross-sectional survey of burnout and provider experiences in ICLs and homecare providers of the ICP using convenience sampling and analyzed the data using mixed methods [30]. Additionally, we conducted a post hoc analysis of semi-structured interviews of ICP staff to further explore provider perspectives on burnout [30]. We selected this mixed methods approach to deepen our understanding of the provider experiences underlying burnout scores and identify promoting and mitigating factors. The ASSESS tool for mixed-methods implementation research [31] was used to guide reporting where applicable.

### 2.3. Data Collection

#### 2.3.1. Institutional Approval

This project received institutional approval as part of a broader evaluation of the effectiveness of the ICP (QIRC 23-0640). All participants were informed about the project and provided ample opportunity to inquire about the evaluation before consenting to participate.

#### 2.3.2. Burnout and Provider Experience Survey

We used the Maslach Burnout Inventory Human Services Survey (MBI-HSS) for Medical Personnel, which includes emotional exhaustion, depersonalization and personal achievement domains, and is the pre-eminent tool for assessing burnout in health care settings like integrated care [12,18]. Using REDCAP hosted at UHN [32], the survey was distributed by email to all 90 staff with patient care roles from December 2024 to February 2025. We used the original version of the MBI-HSS, including 22 questions across 3 subscales representing the 3 domains of burnout [33]. For each question, respondents self-assessed on a Likert scale scored as: Never = 0; A Few Times per Year = 1; Once a Month = 2; A Few Times per Month = 3; Once a Week = 4; A Few Times per Week = 5; and Every Day = 6. Scores in each domain were totaled for each respondent and widely used cut off points [34] for low, moderate and high burnout were applied to indicate respondent-level burnout according to the MBI manual: emotional exhaustion ≤17, 18–29, and ≥30, depersonalization ≤5, 6–11, and ≥12, and personal achievement ≥40, 34–39, and ≤33 [33]. Total scores for each domain were averaged to summarize cohort-level burnout. The MBI was accompanied by demographic questions, including age, gender, duration of professional practice, duration of employment in the ICP, number of patients cared for while working in the ICP and work site (hospital or community). ICLs work at hospital sites, while homecare providers typically work in the community. Due to a low completion rate (86% missingness), respondents’ specific role was excluded from quantitative analysis, but other variables had complete data.

Additionally, there were 5 open-ended questions on workplace experiences identified as priority areas for the ICP: “Please describe how you feel the program has impacted your patients; please describe how you feel the program has impacted your workload; please describe how you feel the program has impacted your overall work satisfaction; please describe how you feel the program has impacted your administrative workflow; and do you have any other comments regarding your experiences in the program which you have not yet shared?”

#### 2.3.3. Provider Experience Interviews

Semi-structured interviews of ICLs, homecare providers, and administrators were conducted as part of a Realist evaluation between November 2023 and April 2024. ICLs were interviewed on their experience working with the ICP, knowledge about the ICP, the program’s impact on patients, communication with patients, quality of work life and any other additional provider experiences. In addition to these questions, homecare providers were also interviewed about the program’s sustainability and ability to support patients with social determinants of health, while administrators were asked about resources and support for ICLs and homecare providers. All interviews were audio-recorded and professionally transcribed verbatim for analysis. Details of this Realist evaluation will be published separately.

### 2.4. Analysis

We used an exploratory QUAN (qual) mixed-methods analysis [30], where open-ended responses in both the survey and provider interviews were used to triangulate burnout determined using MBI scores from the survey. After quantifying levels of burnout, qualitative analysis guided by the MBI was used to explore underlying factors.

#### 2.4.1. Quantitative Analysis

Descriptive statistics were used to summarize demographic variables and burnout. Following an evaluation of data distribution (normality), we explored the impact of respondent characteristics on MBI scores for each domain using Pearson and Spearman rank correlations for continuous variables and Welch’s t tests for categorical variables (R version 4.5.2). Correlation was selected as the preferred method for describing the strength of the relationship between observed variables with the following cut-off points: 0.00–0.10 = negligible, 0.10–0.39 = weak, 0.40–0.69 = moderate, 0.70–0.89 = strong, and 0.90–1.00 = very strong [35]. *p*-values < 0.05 indicated statistical significance.

#### 2.4.2. Qualitative Analysis

We used directed content thematic analysis [36] guided by the multidimensional theory of burnout [19]. Qualitative data was analyzed in NVivo software version 14 (Lumivero, Denver, CO, USA) using an iterative comparative process, with burnout domains as high-level codes. However, an inductive approach was used to generate the lower-level codes from topics that emerged organically from the responses, using MBI questions as coding guides. Due to the workload and over-extension concepts crossing between emotional exhaustion and depersonalization domains, we coded workload under emotional exhaustion, unless there was an explicit mention of emotionally charged interactions with patients or detachment as a coping mechanism, which we coded under depersonalization. We tracked reference counts in NVivo, which correspond to the number of citations for a code, as an indicator of the intensity and corroboration of codes. To identify barriers and facilitators, we analyzed the context of whether statements described promoting or mitigating factors for burnout. We analyzed provider interview transcripts post hoc to further investigate these factors, especially for depersonalization, where reference counts were low in the surveys. After reading initial transcripts, reviewers met to discuss the coding strategy, including definitions and alignment with the MBI. Subsequently, all coding was completed by two reviewers (surveys: A.B., J.O.; interviews: A.A., J.O.) and reviewed by K.O. Through several discussions, we compared codes and references within and between reviewers to develop themes.

Findings from this thematic analysis were interpreted alongside quantitative results and incorporated by J.O. into an integrated analysis guided by the multidimensional theory of burnout [19]. Promoting and mitigating factors would illuminate causes of high and low burnout respectively, and a combination of both would illuminate moderate burnout. Additional validation of the analysis involved presentations to ICP administrators and managers, ICLs, co-investigators, and patient-partners, including a learning health system workshop where burnout-promoting and mitigating factors were discussed to develop future quality improvement strategies.

#### 2.4.3. Positionality

Our team comprised research analysts, a post-doctoral researcher, and a clinician–scientist who were trained in mixed-methods methodology and best practices for implementation evaluation involving human participants. K.O. is also a general internal medicine physician and scientist whose program focuses on care transitions, including new models of care. To limit the impact of researcher viewpoints and preconceptions, our team discussed the research project regularly to maintain consistency in rigor and research practices. More importantly, we also discussed the methodology, analysis and findings with integrated care stakeholders (e.g., ICLs and patient-partners), to incorporate their perspectives and included all co-investigators following the first draft to ensure rigor in analysis and interpretation.

## 3. Results

### 3.1. Respondent Characteristics

Twenty-eight staff responded to the survey, representing a 31% response rate (Table 1). There were 15 staff in community practice, 12 at a hospital site, and one preferred not to answer. Most respondents were women (75%), were 42 ± 10 years old on average and reported medians of 170.0 (IQR = 245.0) patients cared for, 12.0 (12.0) months working in the ICP, and 6.5 (7.8) years of clinical practice.

Twenty staff were interviewed: 13 ICLs, five homecare providers and two administrators. Interviewees were 38 ± 8 years old on average and 95% were women.

### 3.2. Burnout Risk

**Figure 1:**
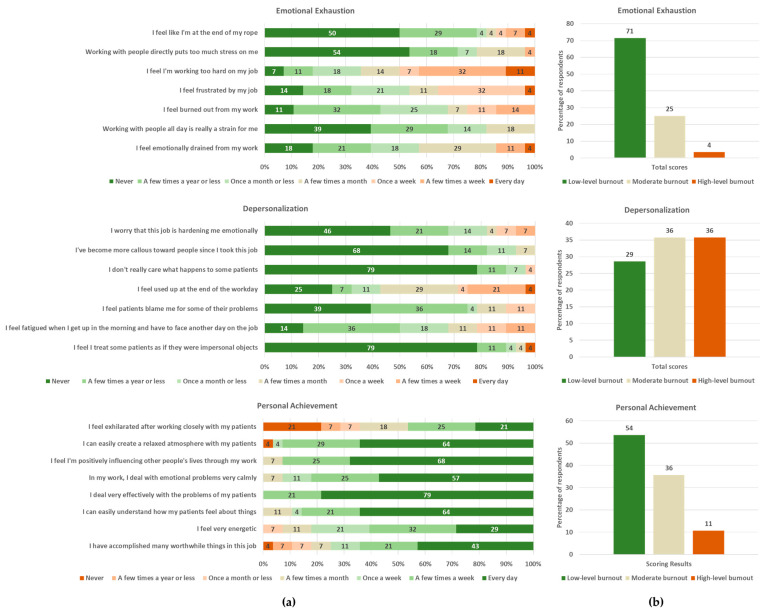
Proportion of responses and burnout for emotional exhaustion, depersonalization and personal achievement: (**a**) percentage of responses on Likert scales; (**b**) percentage of respondents by burnout level. Scores were totaled for each participant, and widely used cut off points for low, moderate and high burnout set according to the manual by Maslach et al. [33]: emotional exhaustion ≤17, 18–29, and ≥30, depersonalization ≤5, 6–11, and ≥12, and personal achievement ≥40, 34–39, and ≤33.

**Figure A1:**
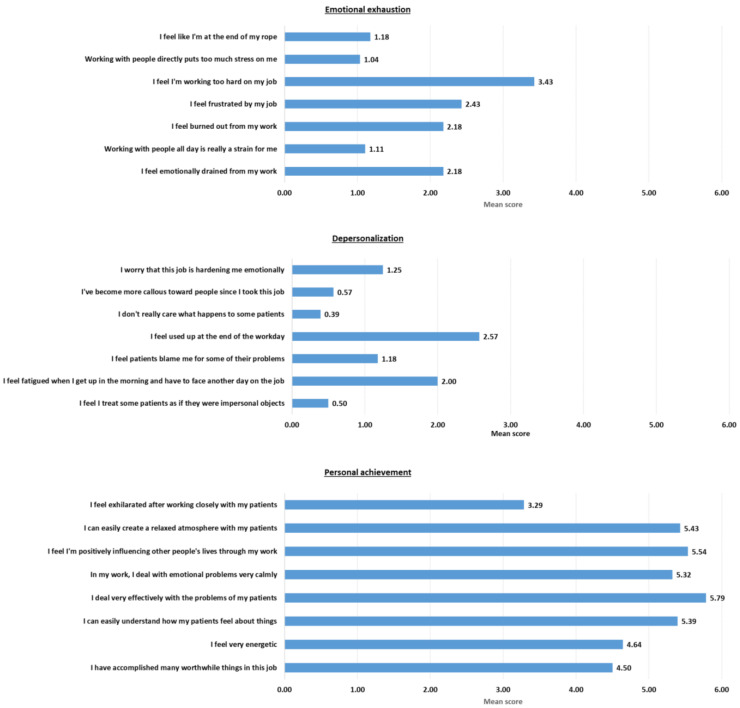
Average MBI scores for each survey question for emotional exhaustion, depersonalization and personal achievement.

The MBI total score averages (±SD) were 13.5 ± 7.3 for emotional exhaustion, 8.5 ± 5.5 for depersonalization, and 39.9 ± 5.1 for personal achievement, indicating low, moderate and low burnout by category, respectively. Detailed proportions of Likert scores by question, as well as proportions of low, moderate and high burnout, are summarized in Figure 1. Average scores by question are summarized in Figure A1. For depersonalization, ‘Feeling used up at the end of the day’ was scored highest on average (Figure A1).

### 3.3. Impact of Survey Respondent Characteristics on Burnout Risk

Working at a hospital site (t = 2.51, *p* = 0.02) and caring for more patients in the ICP (r = 0.48, *p* = 0.01) were significantly associated with higher depersonalization, but not any of the other burnout domains (Table 2). A trend towards moderate correlation was observed between depersonalization and increasing months worked in the ICP (r = 0.41, *p* = 0.05). Conversely, being a woman was marginally associated with less depersonalization (t = −2.27, *p* = 0.05) and emotional exhaustion (t = −1.91, *p* = 0.09). Age and years of professional practice were not significantly associated with any burnout domain. Between MBI domains, depersonalization was strongly correlated with emotional exhaustion (r = 0.75, *p* = 0.03 × 10^−4^), but personal achievement was not correlated with either.

### 3.4. Thematic Analysis of Promoting and Mitigating Factors of Burnout

#### 3.4.1. Emotional Exhaustion

Top-level codes for themes are highlighted in Table A1. Workload emerged as the predominant theme for emotional exhaustion in the survey. Specifically, respondents reported administrative burden as a promoter of burnout, citing duplicative and excessive documentation across different electronic health record systems, as highlighted in the following quote:

*“There is a massive admin burden on ICLs. Having to use 3 different systems that do not talk to each other…more than half a working day can easily be taken up with admin tasks. Adding to burden is the fact we have to input the same information in multiple different places which is time consuming.”*

(Hospital site staff)

Conversely, other survey respondents reported an overall balanced workload as the main mitigating factor for burnout. The flexible work scheduling, which is especially relevant to homecare staff, was ideal for workload management, as stated in the quote below:

*“The workload seems manageable to me although I am not working full time with the Integrated care program…I like the flexibility of being able to choose my hours and see patients at mine and their convenience”*

(Community practice staff)

Similar to the surveys, workload was the most prominent theme in the interviews. Interestingly, most interviewees reported team collaboration as a mitigating factor for emotional exhaustion, with support from colleagues and leadership as crucial in managing their workload, as quoted below:

*“…this is the kind of work that I really want to do and my job satisfaction stems from, you know, the great teamwork, the support that we get from leadership and just learning on a daily basis.”*

(Hospital site staff)

#### 3.4.2. Depersonalization

Coding reference numbers for depersonalization were relatively low (<10) for both the surveys and interviews. Perceived under-appreciation was the leading theme for depersonalization from the survey, with respondents reporting a lack of appreciation for staff as the program size grew, as exemplified below:

*“The program has grown so much that nobody cares anymore about the nurses. They used to appreciate but no longer…The program with has moved my love of the job from the start to now move to part time as I feel unappreciated now”*

(Community practice staff)

On the other hand, depersonalization was mitigated by team support, as survey respondents cited support from coworkers as crucial in managing depersonalization, as quoted below:

*“Overall, the IC team provides a very supportive and positive work environment that helps when dealing with challenging patient situations.”*

(Hospital site staff)

Interestingly, top themes from the interviews differed from the surveys. Emotionally charged contact with dissatisfied patients was the leading depersonalization theme from the interviews; interviewees reported unwarranted blame from patients, as highlighted below:

*“… I thought I was very gentle and respectful, but I don’t know. When I’m thinking about it, I don’t know where I went wrong with them. They weren’t happy. They reported me to the office”*

(Community practice staff)

Personalized care and rapport were the leading mitigating factors for depersonalization according to the interviews. Interviewees cited the need to engage with unique patient needs, which necessitates personalized care and concern for each patient, as noted below:

*“I absolutely love it. Just because as a lead, you have real autonomy to impact and provide literally care to the patients. They’re very personalized and care for the patients that you basically are under your wing, you’re taking care of. Of course, with your team, with your [homecare vendor] team, no, not, of course, alone. In other roles, I find a lot of limits and not necessary, not able to be that directly involved on a bigger scale as I have ability to be involved right now.”*

(Hospital site staff)

#### 3.4.3. Personal Achievement

Patient impact emerged as the predominant theme for personal achievement on both the survey and interviews. The ICP enhanced the respondents’ reach and ability to provide additional support to patients, contributing to improvements in patient health outcomes and the appreciation of staff by patients, as quoted below.

*“For most of my patients, this program has had a positive impact. The enhanced care and access to resources makes a huge difference to their lives and what I can accomplish as a professional.”*

(Community practice staff)

On the other hand, program capacity was the pre-eminent barrier to personal achievement in both the survey and interviews. Survey respondents cited the program enrollment duration and limited allocated time per patient as limiting their ability to support patients, as exemplified below:

*“Workload can be a bit much at times, the amount of time I get paid to see client sometimes is not enough [for the desired] quality of care”*

(Community practice staff)

Similarly, interviewees reported capacity as a main limiting factor for personal achievement, additionally highlighting clinical and social complexity as drivers of unmet needs, as quoted below:

*“So being able to deal with, like, I guess some more of this social aspect is a little harder for us because we don’t have, like, the background in social work to help folks like address like the marginalization issues they may experience.”*

(Hospital site staff)

## 4. Discussion

In this exploratory mixed-methods analysis, we used the MBI to measure burnout among healthcare providers in an ICP that supports hospital-to-home care transitions and identified the underlying promoting and mitigating factors for burnout. Among the domains of burnout, responses indicated low levels of emotional exhaustion, moderate depersonalization and high personal achievement. Having a higher patient load and being primarily hospital-based were associated with higher depersonalization, as was longer work duration in the ICP, albeit marginally. Being a woman was marginally associated with less depersonalization and emotional exhaustion. Respondents cited teamwork and flexible scheduling as the leading contextual factors for low emotional exhaustion, while positive impact on patients was the leading factor for high personal achievement. Under-appreciation and emotionally charged contacts with dissatisfied patients were the main contextual factors of depersonalization, mitigated by team support and a personalized approach to caring for patients.

Healthcare workers experience an elevated risk of emotional exhaustion due to chronic exposure to human distress and long work shifts without adequate rest [34,37], which may be exacerbated by the intense level of personal and emotional contact required in the context of integrated care [11,15,22]. However, multidisciplinary ‘team-based’ models of care like integrated care also confer opportunities to manage burnout through directed and shared responsibility with flexibility and workload distribution [20,38,39]. In our ICP, ICLs work with the inpatient team, homecare providers and other ICP staff as a team mobilized around unique patient care needs. Therefore, we speculate that emotional exhaustion was low due to team support from colleagues and leadership, which were identified as mitigating factors. ICLs cited institutional support, such as effective administrators and managers, to be crucial in creating a conducive environment, while homecare staff cited flexible work arrangements, such as remote work and choosing their work hours. Organizational support, effective teamwork and flexibility have been shown to decrease burnout in integrated care [12,15,20,39]. Interestingly, a trend towards lower emotional exhaustion was observed in women. In our team of predominantly women, this [19] may reflect the impact of organization-specific dynamics of gender composition [40], which affect burnout among healthcare providers [41]. This effect ought to be interpreted cautiously since it was only marginally statistically significant in our cohort (confidence interval crosses zero); further research is warranted given mixed but limited findings from other healthcare studies [42,43,44,45]. Like others [25,46], administrative tasks were a prominent sub-theme in our qualitative analysis, indicating room for improvement to sustain the low risk of emotional exhaustion. Specifically, integration of electronic health records systems could improve cross-team collaboration, bridging hospital and home care records [12,47,48]. Our observations contrast with other healthcare settings, where emotional exhaustion is high [34,46,49], and highlight the strength of this team-based model of care.

Similar to emotional exhaustion, the personal achievement domain indicated low burnout in our cohort. Given the intense multidisciplinary engagement required to address crucial gaps in care transition [11], this ICP offers additional opportunities for healthcare providers to not only connect effectively with patients but also ensure and track the impact on patients. Based on identifying positive patient health outcomes and experiences as the top promoting factors, we speculate that personal achievement was facilitated as staff were empowered to provide more comprehensive support and observe its impact. Our findings are consistent with high personal achievement scores in other ICPs [12,24], with personal achievement being enhanced by greater integration [21].

Depersonalization in our cohort was moderate overall, with an equal proportion of respondents reporting moderate and high total scores in the depersonalization domain (Figure 1b). This is consistent with findings from a comparative study of low, partial and high uptake ICPs, where moderate depersonalization was observed in the partial uptake group characterized by shortcomings in either leadership or turnover [20]. In addition to loss of idealism, withdrawal, cynicism, and callousness towards patients, depersonalization is also linked to chronic fatigue [19]. Based on our MBI survey, ‘feeling used up’ had the highest average score in the depersonalization domain, highlighting an element of chronic fatigue that may be shared with emotional exhaustion. This may explain its strong correlation with emotional exhaustion in our evaluation—a relationship that has long been recognized [19]. Moreover, the positive association with patient load, as well as the trend with duration worked in the ICP in our evaluation, is consistent with the link between workload and depersonalization corroborated by others [46,49,50,51]. Notably, these effects are relatively small in magnitude, with lower confidence intervals approaching zero, and thus, warrant future replication. Interestingly, while Zubatsky et al. reported a decrease in depersonalization with increasing care integration in behavioral healthcare providers, they also reported a decrease in depersonalization after ≥10 years of employment [21]. This trend could reflect differences between ICPs and/or the differential effect of employment duration beyond the relatively short period in our evaluation.

Working at a hospital site, which is necessary for the ICL role but not homecare providers, was associated with higher depersonalization. This effect was greater in magnitude than that of other workplace factors, which may indicate a greater relative role. Exploration of the workload theme revealed that flexible scheduling and remote work, which are available to the homecare staff but not ICLs, were mitigating factors for burnout. We speculate this is because they offer greater control over workload for a better work–life balance. This is corroborated by lower depersonalization in community nurses compared to hospital nurses in another study [52], and is consistent with the job Demand–Control–Support model, which links job demands and control within one’s role to burnout [53]. Being a woman was marginally associated with less depersonalization in our evaluation, which is similar to earlier research [19,42,45]. This might be explained by a tendency for men to more readily use emotional detachment as a protective mechanism under stress [42]. However, this effect ought to be interpreted cautiously for the same reasons stated above for emotional exhaustion and warrants further research. Under-appreciation and emotionally charged contacts with patients emerged from qualitative analysis as depersonalization-specific promoters of burnout from the survey and interviews respectively. Given the timing of the interviews relative to the surveys, we speculate that challenging patient encounters were an early challenge of the ICP that was ameliorated by increased team support from a growing collaborative workforce. ICPs decrease emotionally charged contacts with dissatisfied patients [22], which may explain the differences in the interviews and survey themes as the program matured. Moreover, as the program grows, there may be a need to be more deliberate in highlighting the essential contributions of all multidisciplinary providers to help improve appreciation for all roles. Qualitative analysis for depersonalization ought to be interpreted cautiously, however, since reference counts were low. In the future, a more focused, direct exploration of depersonalization through semi-structured interviews could elucidate this phenomenon.

### 4.1. Strengths and Limitations

To the best of our knowledge, this is the first mixed-methods exploration of burnout using the MBI in integrated care coordinators and homecare providers for a hospital-to-home transition program. The mixed-methods approach guided by the multidimensional theory of burnout aligns our analysis with the gold standard for burnout assessment. However, our evaluation has some limitations. Since this was a cross-sectional evaluation without a comparator group, causal relationships for burnout could not be inferred. Moreover, levels of burnout were inferred from the MBI manual and the published literature, not clinical assessment. The relatively small sample size reduced statistical power and made our estimates of burnout more susceptible to random variation. However, the qualitative responses provided valuable depth and context to support the interpretation of findings. Secondly, convenience sampling could have introduced a self-selection bias in this sample; survey participation was not mandatory and burnout was self-reported. Additionally, the 31% response rate was lower than in other burnout studies on integrated care coordinators [12] and homecare providers [54], and thus increased the risk of non-response bias. Moreover, most survey respondents did not indicate their roles, which limited our investigation into the impact of roles on burnout. Individuals with high burnout may be less likely to participate, or conversely, more motivated to respond. The lack of non-respondent data also limits our ability to assess differences between respondents and non-respondents. Additionally, this evaluation was conducted at a single hospital network on a sample of predominantly female respondents. Therefore, generalizability within and beyond our project may be limited, which future studies in integrated care should consider. As this was an exploratory evaluation, we did not adjust for confounding factors for the relationship between respondent characteristics and burnout. Lastly, the post hoc use of provider interview data for secondary analysis limited the ability of our qualitative analysis to fully explain the quantitative findings. Moreover, while coding was guided by MBI concepts, open-ended questions in the survey and interview guides were not directly aligned with MBI questions, therefore limiting the integration of qualitative and quantitative findings. Given the important limitations above, our work may be important in generating hypotheses and further depth for future research in integrated care.

### 4.2. Future Directions

Future studies ought to investigate burnout longitudinally before and after integrated care interventions or following significant changes within integrated care implementation. Larger sample sizes and higher response rates, achieved through greater engagement and knowledge mobilization with the integrated care staff, would improve the robustness of the analyses. As integrated care scales and transforms care delivery, assessing burnout of inpatient staff broadly might elucidate its institutional impact as care transition responsibilities are shifted and post-discharge hospital visits are averted. Future research should also incorporate empirically supported person–job imbalance models that are closely linked with the MBI, such as the Areas of Work–life model, which identifies imbalances in workload, control, values, reward, fairness and community [50]. This could be enhanced by job satisfaction and lifestyle impact surveys that indicate the downstream effects of burnout [49]. All future research will also benefit from standardizing burnout assessment methodology, especially when using the MBI, to facilitate comparisons across studies [34]. Lastly, a priori incorporation of qualitative methods, such as interviews, aligned with the MBI would better explain the co-factors of burnout.

## 5. Conclusions

As ICPs are implemented to meet the growing demand for patient-oriented care, there is a growing need to investigate healthcare provider burnout as a program sustainability measure. In a sample of ICP healthcare providers supporting hospital-to-home transitions, burnout risk was low to moderate—emotional exhaustion was low and personal achievement was high, with effective workload management and positive impact on patients as the prominent contextual factors respectively. Depersonalization was moderate, with tentative positive associations with working at a hospital site and caring for a greater number of patients while working in the ICP. Perceived under-appreciation and challenging patient interactions were leading contextual factors for depersonalization overall, and teamwork and rapport were mitigating factors. These findings indicate the continuing need for ICPs to foster supportive team environments for staff and manage workloads to mitigate depersonalization and sustain the positive impact on patients, which in turn confers a sense of accomplishment and purpose to healthcare providers. Overall, we highlight strengths, as well as critical areas of improvement for integrated care implementation that warrant validation in future studies.

## Figures and Tables

**Table 1:** Demographic and workplace characteristics.

Characteristics	Survey	Interviews
Age, mean ± SD	42.3 ± 9.7	38.0 ± 8.4
Gender, N (%)		
Woman	21 (75)	19 (95)
Man	7 (25)	1 (5)
Work location, N (%)		
Hospital site	12 (43)	13 (65)
Community practice	15 (54)	7 (35)
Unknown	1 (4)	0 (0)
Months in Integrated Care, median [IQR]	12.0 [12.0]	N/A ^1^
Patients cared for, median [IQR]	170.0 [245.0]	N/A ^1^
Years practiced, median [IQR]	6.5 [7.8]	N/A ^1^

^1^ Data was not available. It was not collected for interviewees.

**Table 2:** Relationships between burnout domains and survey respondent characteristics.

Variable	Emotional Exhaustion	Depersonalization	Personal Achievement
Age ^1^	0.04 (−0.34, 0.41)	−0.10 (−0.46, 0.28)	0.23 (−0.16, 0.56)
Gender: woman ^2^	−1.91 (−13.84, 1.18) *	−2.27 (−12.21, 0.11) *	0.43 (−3.25, 4.87)
Work site: hospital ^2^	0.74 (−3.64, 7.70)	2.51 (0.84, 8.66) **	−1.10 (−6.10, 1.86)
Months worked in the ICP ^3^	0.20 (−0.19, 0.53)	0.37 (0.00, 0.65) *	−0.03, (−0.39, 0.35)
Patients cared for while working in the ICP ^3^	0.27 (−0.13, 0.67)	0.48 (0.15, 0.82) **	0 (−0.46, 0.46)
Years of professional practice ^3^	−0.07 (−0.50, 0.36)	−0.05 (−0.49, 0.39)	0.18 (−0.29, 0.66)
Emotional exhaustion ^1^	-	0.75 (0.53, 0.88) **	−0.30 (−0.60, 0.09)
Depersonalization ^1^	0.75 (0.53, 0.88) **	-	−0.31 (−0.61, 0.07)
Personal achievement ^1^	−0.30 (−0.60, 0.09)	−0.31 (−0.61, 0.07)	-

^1^ Pearson correlation; ^2^ Welch’s t test; ^3^ Spearman rank correlation; ** *p* value < 0.05; * *p* value ≥ 0.05–0.1.

## Data Availability

The raw data supporting the conclusions of this article will be made available by the authors on request.

## Abbreviations

The following abbreviations are used in this manuscript:

ICL	Integrated Care Lead
ICP	Integrated Care Program
MBI	Maslach Burnout Inventory
MBI-HSS	Maslach Burnout Inventory Human Services Survey
UHN	University Health Network

## Appendix A

**Table A1:** Contextual factors for burnout.

Burnout Category	Context	Provider Survey (*n* ^1^)	Provider Interviews (*n* ^1^)
Emotional exhaustion	Promoting factors	Excessive workload (39) Procurement & supplies (3) Remuneration (2)	Excessive workload (55) Teamwork challenges (26) Technology (7)
	Mitigating factors	Balanced workload (12) Team cooperation (3) Technological supports (3)	Team cooperation (36) Balanced workload (15)
Depersonalization	Promoting factors	Under-appreciation (3) Challenging patients (1)	Challenging patients (4) Under-appreciation (1)
	Mitigating factors	Team support (3)	Personalized patient care (4) Patient rapport (2)
Personal achievement	Limiting factors	Program capacity (3) Limited growth opportunities (2)	Program capacity (117) Teamwork challenges (21)
	Promoting factors	Patient impact (24) Patient feedback (7) Personal development (6)	Patient impact (96) Team collaboration (25) Personal development (23)

^1^ Number of respondent/interviewee statements cited for underlying codes—an indicator of the intensity and corroboration.
